# RAB27B inhibits proliferation and promotes apoptosis of leukemic cells via 3-Hydroxy butyrate dehydrogenase 2 (BDH2)

**DOI:** 10.1080/21655979.2022.2036903

**Published:** 2022-02-14

**Authors:** Can Meng, Li Huang, Xiangjun Fu, Bin Wu, Lie Lin

**Affiliations:** Department of Hematology, Hainan General Hospital (Hainan Affiliated Hospital of Hainan Medical University), Haikou City, Hainan, China

**Keywords:** RAB27B, BDH2, acute myeloid leukemia

## Abstract

RAB27B is a member of Ras-like small GTPases that plays a role in endocytosis, exocytosis, and vesicle trafficking. We made an attempt to study the impacts of RAB27B on the proliferation and apoptosis of acute myeloid leukemia (AML) cells. The silencing of RAB27B was induced by siRNA for the detection of proliferation, cell cycle, and apoptosis, respectively by Cell Counting Kit-8 (CCK8), flow cytometry, and TUNEL. Related markers were also evaluated by Western blot analysis. The interaction between RAB27B and BDH2 was predicted by bioinformatics analysis and determined by immunoprecipitation. The gain of function of BDH2 was also detected by these functional assays. RAB27B exhibited high levels in AML cells, and RAB27B silencing led to reduced proliferation, increased cell cycle arrest and apoptosis levels. Then, the interaction between RAB27B and BDH2 was confirmed. Moreover, the effects of RAB27B inhibition on the proliferation, cell cycle arrest, and cell apoptosis were abolished after BDH2 overexpression. RAB27B inhibits proliferation and promotes apoptosis of leukemic cells by interacting with BDH2. Targeting RAB27B might be an effective method for the treatment of AML.

## Graphical Abstract

**Figure:**
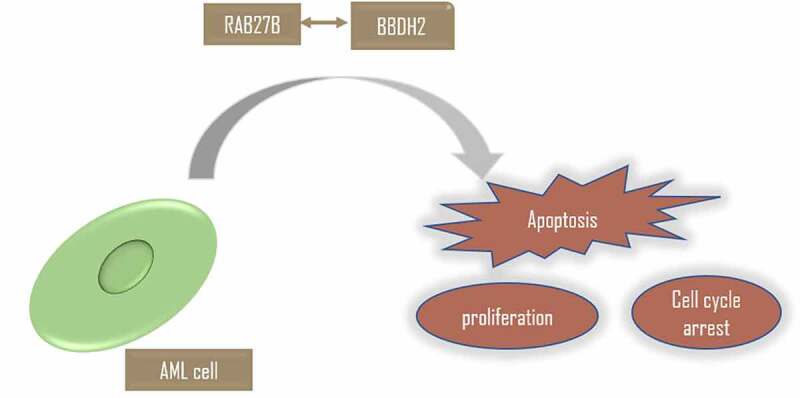

## Introduction

Acute myeloid leukemia (AML) is a genetically heterogeneous disease featured by the significantly rapid growth of abnormal myeloid progenitor cells in the bone marrow and blood that can interfere the function of normal blood cells [1,2]. As for the occurrence of AML, it starts from the step-wise aggregation of genetic alterations influencing proliferation and/or differentiation in hematopoietic stem or progenitor cells [3]. Patients with AML show equivocal weight loss, fever, thrombosis, or anemia, low immunity, or blood loss [4]. The median overall survival of AML patients that are 18–60 years old is approximately 40%, which is indicative of the treatment of AML for the family [5]. Amounts of studies have recognized some biological molecules involved in the progression of AML, which are found to have potential therapy and diagnosis effects for AML [6–8]. Regardless of significant progress that has achieved in the molecular diagnosis and treatment of AML, the pathogenesis of AML is still poorly illustrated.

The Rab family is a type of Ras-like small GTPases that plays a role in endocytosis, exocytosis, and vesicle trafficking [9,10]. RAB27B, a member of the Rab group, has been widely shown to play a crucial role in facilitating cancer progression and metastasis [11]. Moreover, it was closely associated with the malignant behaviors of tumor cells [12]. Intriguingly, a line of evidence has demonstrated the inhibition of RAB27B by miR-34c-5p induces the senescence of AML stem cells, firstly linking the RAB27B expression to AML development [13]. Nevertheless, there is a shortage of investigations into the role of RAB27B in the proliferation and apoptosis of AML stem cells.

BDH2, originally named DHRS6, is recognized as a member of the short-chain dehydrogenase/reductase family [14]. Its expression can be ubiquitously observed in cytoplasm of epithelial cells in multiple organs including the kidney, small intestines, and breast [15]. Serving as a cytosolic-type 2-hydroxybutyrate dehydrogenase, it is capable of physiologically modulating the cytosolic ketone bodies [14]. The association between BDH2 and AML was firstly discussed by a previous study that implied BDH2 as an independent poor prognostic factor, which plays an anti-apoptotic role in cytogenetically normal AML [16].

We assume that there exists an association between RAB27B and BDH2, which is involved in the progression of AML. In the present study, we explored the pivotal role of RAB27B in AML and discussed the potential association between RAB27B and BDH2. Moreover, we conducted a series of tests for the validation of our hypothesis that RAB27B might modulate the proliferation and apoptosis of leukemic cells via targeting BDH2.

## Materials and methods

### Cell culture

AML cell lines KG-1 and human bone marrow stromal cell line (HS-5) were purchased from American Type Culture Collection (Manassas, VA, USA). AML-193 cells were obtained from Procell Life Science&Technology Co., Ltd (Wuhan, China), while K-652 cells from Kunming Cell Bank (KCB, Kunming, China). AML cells were cultured in Iscove’s Modified Dulbecco’s Medium (Thermo Fisher Scientific, Waltham, MA, USA) with 10% FBS (Gibco, Gran Island, NY, USA) and 1% penicillin/streptomycin (Beyotime, Shanghai, China) in an atmosphere with 5% CO_2_ at 37°C. HS-5 cells were placed in Dulbecco’s Modified Eagle’s Medium (Thermo Fisher Scientific) added with 10% FBS and 1% penicillin/streptomycin under the same condition.

### Western blotting assay

Total protein was isolated from AML cells, and then a BCA Protein Assay Kit (Beyotime Biotechnology) was used to measure protein concentration. The samples were separated by electrophoresing on 10% SDS-PAGE and then transferred to PVDF membranes. Possible nonspecific binding was sealed by 5% nonfat milk and the proteins were then incubated overnight at 4°C with specific primary antibodies (RAB27B, ab76779, 1:1000; Ki67, ab92742, 1:5000; PCNA, ab92552, 1:2000; CDK21, ab227662, 1:400; p21, ab109520, 1:2000; Bcl-2, ab32124, 1:1000; Bax, ab182733, 1:1000; cleaved caspase3, ab2302, 1:500; cleaved PARP, ab32064, 1:1000; BDH2, ab254710, 1:1000; GAPDH, ab8245, 1:5000. Abcam, England). After rinsing with TBST for three times, these membranes were incubated with secondary antibody (ab7068, 1:10,000, Abcam, England). The bands were visualized using an enhanced chemiluminescence detection system (Life Technologies, Pleasanton, CA, USA). GAPDH served as the protein-loading control.

### RT-qPCR

Total RNA was isolated using TRIzol® reagent (Invitrogen; Thermo Fisher Scientific, Inc.) based on the manufacturer’s protocol. RNA was synthesized into cDNA by the SuperScript IV First-Strand Synthesis System (Thermo Fisher Scientific) under the following conditions: 25°C for 10 min, 37°C for 120 min, and 85°C for 5 min. cDNA was used for real-time PCR conducted by the Power SYBR Green Master Mix (cat. no. 4,367,659; Applied Biosystems, Foster City, CA, USA) on a 7300 Real-Time PCR System (Applied Biosystems). All values were normalized to GAPDH, and the calculation of the fold-change was conducted by 2^−ΔΔCq^ method [17]. The primers used are as following: RAB27B forward 5′-TAGACTTTCGGGAAAAACGTGTG-3′, reverse 5′-AGAAGCTCTGTTGACTGGTGA-3′; BDH2 forward 5′- GCTTCCAGCGTCAAAGGAGTT-3′, reverse 5′- CAGTTGCGAATCTTCCCGTC-3′; GAPDH forward 5′- GGAGCGAGATCCCTCCAAAAT −3′, reverse 5′- GGCTGTTGTCATACTTCTCATGG-3′.

## Plasmid transfection

SiRNA targeting RAB27B or siRNA-NC (5′- CAGUAGGAAUAGACUUUCG dTdT-3′, 3′-dTdTGUCAUCCUUAUCUGAAAGC-5′), BDH2 overexpressing plasmids or empty plasmid (PcDNA3.1) were constructed and purchased from Genomeditech (Shanghai, China) transfected into AML cell using LipofectamineTM2000 with the manufacturer’s instructions (Invitrogen, USA). After 24 h, cells were used for further experiment.

CCK-8 assay

For the detection of cell viability, the cells were seeded into 96-well plates at a concentration of 7500 cells/well and 10 μL CCK-8 reagent was added to each well at indicated time points. After that, the cells were incubated with normal cell culture medium for 2 h, and the absorbance was detected at 450 nm by a microplate reader.

### Flow cytometry

The transfected cells were harvested at 72 h and treated with 0.25% trypsin. Then, the cells were centrifuged in order to dump the supernatant. After being fixed by pre-cooled 70% ethanol solution and incubated at 4°C overnight, cells were washed for three times by PBS on the following day. Subsequently, cells were double-stained with Annexin VFITC and propidium iodide (PI), and then the cell cycle was determined. Data analyses were performed by FlowJo software (Tree Star, Ashland, OR, USA).

Terminal DNA transferase-mediated dUTP nick end labeling (TUNEL) assay

TUNEL was conducted using in situ cell death detection kits TUNEL (KeyGen, Biotechnology, Co., LTD, China) according to the manufacturer’s protocol. DAPI (4,6-diamidino-2-phenylindole) was used for the counterstaining of cell nuclei. The number of TUNEL-positive cells was calculated randomly for three visions under an inverted microscope.

### Immunoprecipitation

The cells were lysed by immunoprecipitation lysis buffer (Thermo Fisher Scientific) containing protease inhibitors and phosphatase inhibitors cocktail (Sigma-Aldrich). Cell lysates were then treated with anti-RAB27B or anti-BDH2 antibody and protein A/G beads (Thermo Fisher Scientific) at 4°C for 6 h. After being washed by immunoprecipitation lysis buffer, the beads were boiled for 5 min with 2× SDS loading buffer. Proteins were then analyzed by Western blot.

### Statistical analysis

GraphPad Prism software (version 8.0; GraphPad Software, Inc.) was applied for data analysis. Data were expressed as the mean ± standard deviation (SD). The comparisons between the two independent groups were conducted by Student’s t-test, while the data among multiple groups were analyzed using one-way ANOVA followed by Tukey’s post hoc test. P < 0.05 was considered to indicate a statistically significant difference.

## Results

### RAB27B was increased in AML patients

**Figure 1.**
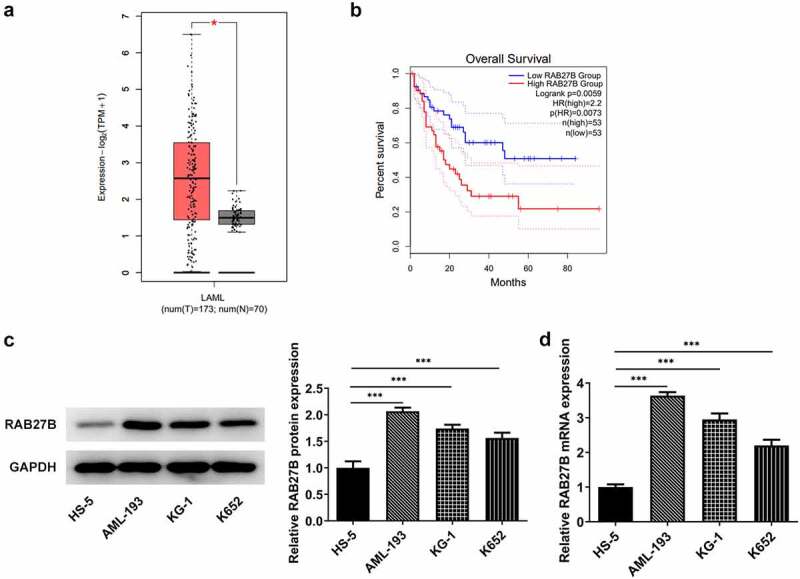
RAB27B was increased in AML patients. (a-b) The expression of RAB27B and overall survival in AML patients. *P < 0.05. (c-d) The protein and mRNA levels of RAB27B in AML cells. The results are representative of four independent experiments. The data were shown in mean ± SD.***P < 0.001.

To analyze the role of RAB27B in AML and discover the potential mechanism, we firstly hunted for the expression of RAB27B in AML by GEPIA database (http://gepia.cancer-pku.cn/) [18], where we found that RAB27B presented abnormally high expression in AML patients (Figure 1a). Furthermore, higher expression of RAB27B was accompanied by poorer prognosis of AML patients by survival analysis using GEPIA database (Figure 1b). Then, we determined the expression of RAB27B in AML cells by RT-qPCR and Western blot. As shown in Figure 1c-D, significant increases in the expression of RAB27B were observed, and AML-193 cells that exhibited the highest RAB27B level were applied for further assays.

### Suppression of RAB27B reduced the proliferation of AML cells

**Figure 2.**
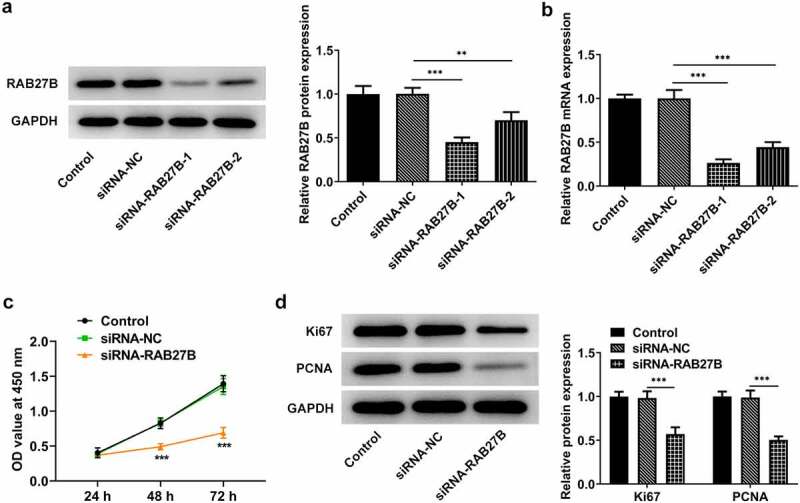
Suppression of RAB27B reduced the proliferation of AML cells. (a-b) The protein and mRNA levels of RAB27B after transfection by Western blot and RT-qPCR analysis. (c)The OD value at 450 nm by CCK8 assay and (d) expression of Ki67 and PCNA after transfection of siRNA-RAB27B-1, siRNA-RAB27B-2 or siRNA-NC for 24 h by Western blot analysis. Each experiment was repeated at least three times. The data were shown in mean ± SD. **P < 0.01, ***P < 0.001.

As RAB27B was high expressed in AML, we next silenced RAB27B to see if the malignant behaviors of AML cells were transformed. Results in Figure 2a-b illustrated that siRNA-RAB27B-1 downregulated the expression of RAB27B at a lower level. Thus, siRNA-RAB27B-1 was used for the following experiments. As for the cell proliferation, the OD value at 450 nm in the siRNA-RAB27B group was evidently lower than that in Control or siRNA-NC group, suggesting that the proliferation of AML-193 cells was reduced in AML-193 cells transfected with siRNA-RAB27B (Figure 2c). Meanwhile, the expression of Ki67 and PCNA was also suppressed by knockdown of RAB27B (Figure 2d). Taken together, these data suggested that inhibition of RAB27B reduced the proliferation of AML cells.

### Suppression of RAB27B promoted the cell cycle arrest at G0/G1 phase and the cell apoptosis

**Figure 3.**
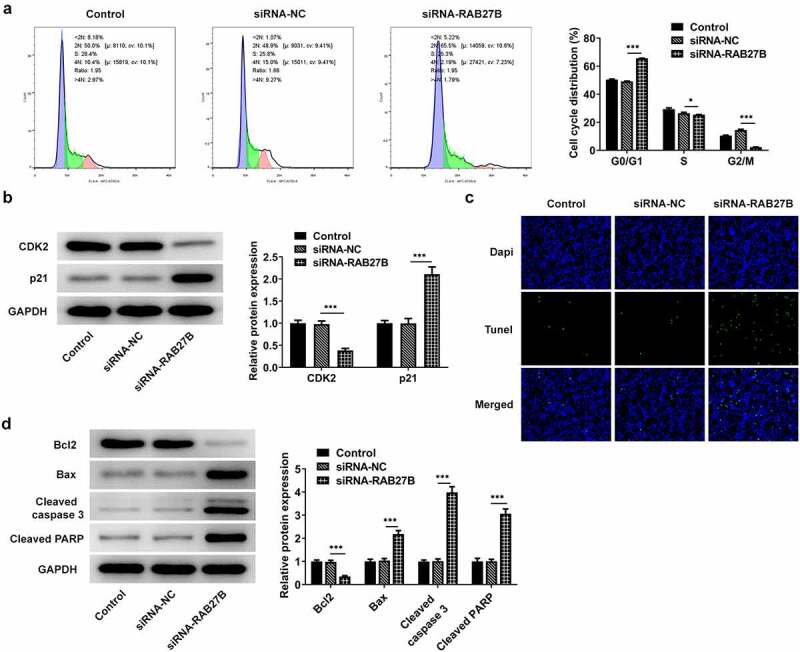
Suppression of RAB27B promoted the cell cycle arrest at G0/G1 phase and the cell apoptosis. (a-b) Cell cycle and expression of CDK2 and p21 were determined in AML cells targeted by si-RAB27B by flow cytometry and Western blot analysis. (c-d) The cell apoptosis and apoptosis-related markers were determined in AML cells targeted by si-RAB27B by TUNEL staining and Western blot analysis. Each experiment was repeated at least three times. The data were shown in mean ± SD. ***P < 0.001.

RAB27B has been reported to regulate apoptosis in some cancers [11,19,20]. We next wonder to know whether and how RAB27B affects apoptosis in AML-193 cells. Then, the cell cycle and apoptosis of AML-193 cells transfected with siRNA-RAB27B were determined to reveal whether the cell cycle and apoptosis could be transformed. Cell cycle analysis showed that silencing of RAB27B triggered obvious increase in the G0/G1 phase and decrease in the G2/M phase (Figure 3a). Meanwhile, it led to lower CDK2 expression and higher p21 expression (Figure 3b). As comparison to the siRNA-NC group, TUNEL analysis showed more TUNEL-positive AML-193 cells in siRNA-RAB27B group, together with downregulated anti-apoptotic Bcl2 expression and pro-apoptotic Bax, cleaved caspase 3 and cleaved PARP expression (Figure 3c-d). These data together implied the role of RAB27B in regulating the cell cycle and apoptosis of AML-193 cells.

### RAB27B could bind to BDH2

**Figure 4.**
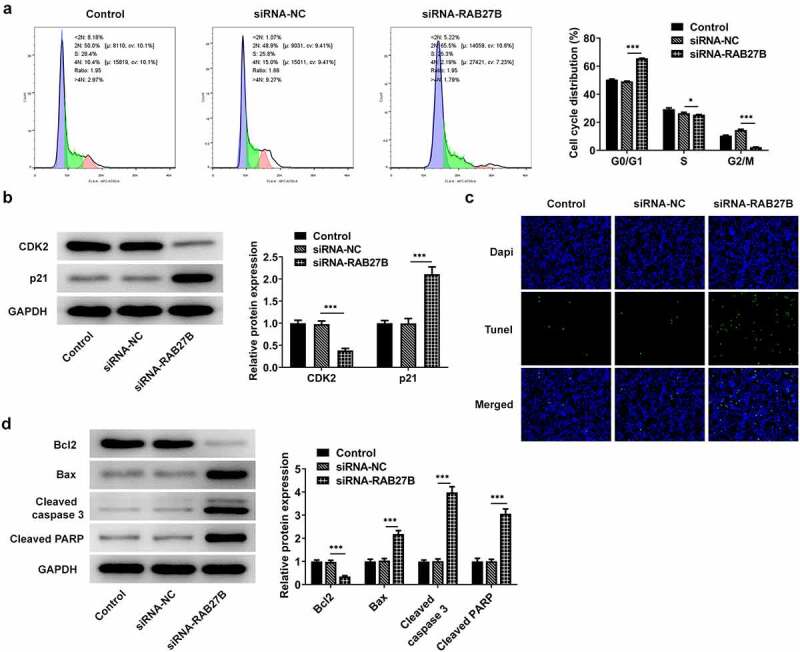
RAB27B could combine with BDH2. (a) The expression of BDH2 in AML cells by the detection of RT-qPCR. (b) The targeting relationship between RAB27B and BDH2 was confirmed by IP. (c) The expression of BDH2 in AML cells targeted by si-RAB27B by Western blot analysis. Each experiment was repeated at least three times. The data were shown in mean ± SD. ^* **^P < 0.001.

The anti-apoptotic factor BDH2 was shown to express at high level in AML, and there is a link between BDH2 and poor prognosis of AML patients. Thus, we attempted to get a clue regarding the role of BDH2 in AML cells. Prediction from BIOGRID tool revealed that RAB27B could bind to BDH2. To further analyze the underlying mechanism, we conducted RT-qPCR and found high expression of BDH2 in AML cells (Figure 4a). Furthermore, RIP assay confirmed the interaction between RAB27B and BDH2 (Figure 4b). Next, we silenced the expression of RAB27B and observed lower mRNA and protein levels of BDH2 in Figure 4c, which indicated their positive association.

### RAB27B suppressed the proliferation of AML-193 cells by binding to BDH2

**Figure 5.**
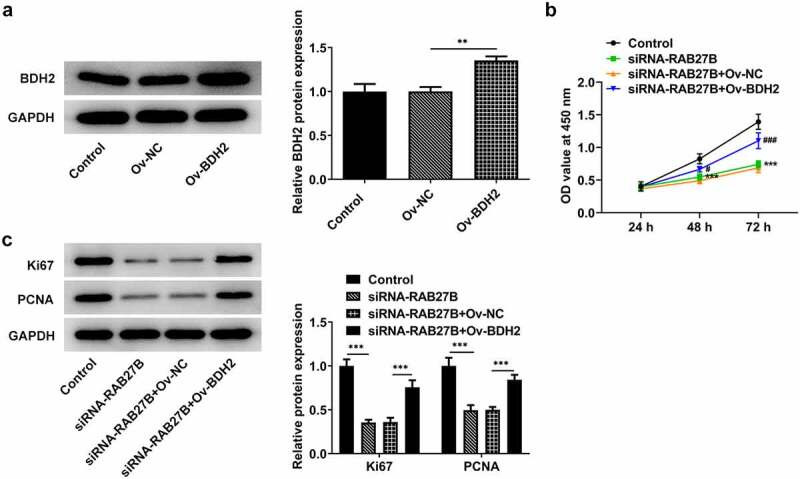
RAB27B suppressed the proliferation of AML-193 cells by binding to BDH2. (a) The protein levels of BDH2after transfection of Ov-BDH2. (b) The OD value at 450 nm by the analysis of CCK8 assay and (c) expression of Ki67 and PCNA after co-transfection. Each experiment was repeated at least three times. The data were shown in mean ± SD. **P < 0.01, ***P < 0.001.

To investigate the potential mechanism by which RAB27B suppressed the proliferation of AML-193 cells, we overexpressed the expression of BDH2. Successful transfection was observed in Figure 5a. Compared with the siRNA-RAB27B+Ov-NC group, the proliferation of AML-193 cells transfected with siRNA-RAB27B and Ov-BDH2 was remarkably increased, as suggested by the elevated OD value at 450 nm (Figure 5b). Moreover, the proliferation-related markers, Ki67 and PCNA, exhibited higher levels after co-transfection with siRNA-RAB27B and Ov-BDH2 (Figure 5c). Thus, these results indicated that RAB27B suppressed the proliferation of AML-193 cells by binding to BDH2.

Suppression of RAB27B promoted the cell cycle arrest at G0/G1 phase and the cell apoptosis by binding to BDH2

**Figure 6.**
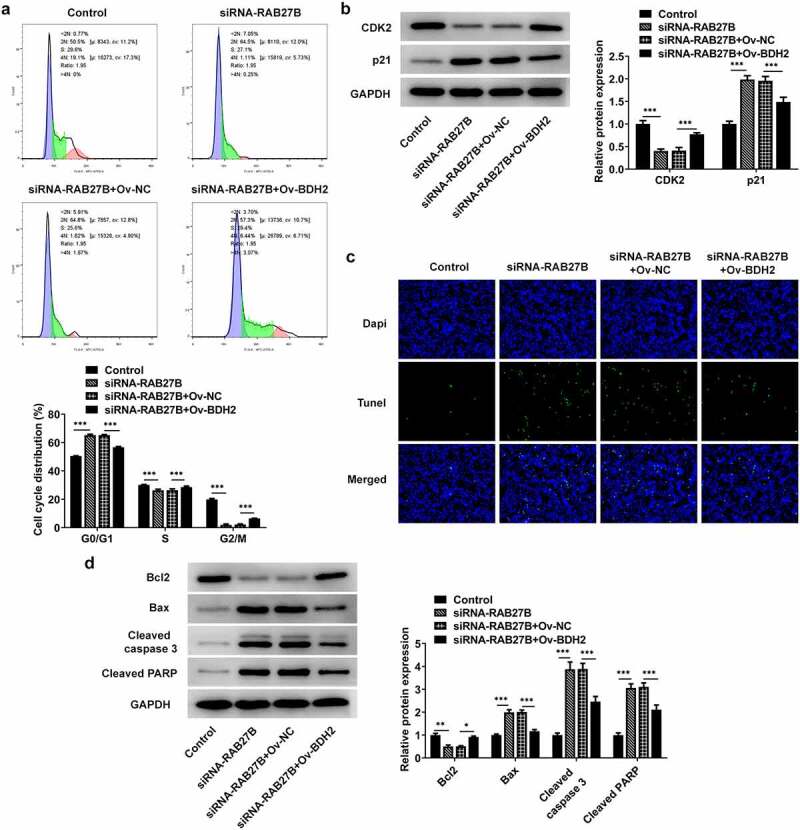
Suppression of RAB27B promoted the cell cycle arrest at G0/G1 phase and the cell apoptosis by binding to BDH2. (a-b) Cell cycle by the analysis of flow cytometry and expression of CDK2 and p21 were determined in AML cells after co-transfection. (c-d) The cell apoptosis and apoptosis-related markers were determined in AML cells after co-transfection. Each experiment was repeated at least three times. The data were shown in mean ± SD. *P < 0.05, **P < 0.01 and ***P < 0.001.

Then, we analyzed whether RAB27B silencing did effects on the cell cycle and apoptosis of AML-193 cells by binding to BDH2. RAB27B knockdown induced an obvious increase in the cells of G0/G1 and a decrease in the G2/M population, which was reversed by BDH2 overexpression (Figure 6a). Additionally, the expression of CDK2 and p21, respectively, decreased and increased by RAB27B knockdown was reversed by BDH2 overexpression (Figure 6b). The sharply increased number of TUNEL-positive AML-193 cells induced by RAB27B knockdown was decreased by BDH2 overexpression, suggesting the apoptosis of AML-193 cells increased by RAB27B silencing was suppressed by BDH2 overexpression (Figure 6c-D). Thus, the suppression of RAB27B promoted the cell cycle arrest at G0/G1 phase and the cell apoptosis by binding to BDH2.

### Discussion

In this paper, the prediction from GEPIA tool indicated that the abnormally high expression of RAB27B was possibly correlated with the poor prognosis of AML patients. Concurrently, we determined the expression of RAB27B in AML cells, finding the elevated expression of RAB27B in various AML cells. Therefore, RAB27B may be an independent diagnostic and prognostic marker of AML.

RAB27B, primarily recognized within platelets, was implicated in exosome secretion [21]. Recent investigations mainly focused on the role of RAB27B in cancers, such as breast cancer, bladder cancer, and glioma [22,23]. Mechanistically, the RAB27B expression was linked to lymph node metastasis and differentiation in breast cancer [22]. The clue from one report elucidating the aberrant expression in AML supported our speculation that RAB27B might exert crucial impacts on the AML cell fate.

Since AML is a heterogeneous malignancy characterized by immature clonal myeloid cell proliferation and abnormal differentiation, many studies have identified cell proliferation inhibition targets for AML therapy [24]. LncRNA NR-104098 serves as a suppressor gene and plays an essential role in inhibiting the proliferation of AML cells [25]. The depletion of GATA2 incurred decreased proliferation and increased apoptosis in AML cells, which suggested the potential application of GATA2 targets as a therapeutic method for AML [26]. TLR4 inhibitor TAK-242 was deemed as an effective treatment strategy against AML owing to its inhibition of AML cell proliferation [27]. Herein, due to RAB27B was high expressed in AML cells, we silenced the expression of RAB27B and detected if its proliferation was changed. The escalated OD value at 450 nm and downregulated expression of proliferation markers Ki67 and PCNA indicated that the proliferation of AML cells was suppressed by RAB27B knockdown. Current evidence has revealed that the combination of cell-cycle active agents with targeted agents may identify successful clinical application for AML treatment [28]. This view of point was congruent with the finding in this study that the cell cycle arrest was induced by RAB27B inhibition.

BDH2 was found to play a key role in the pathogenesis of various cancers [15]. Congruent with previous study indicating that BDH2 was highly expressed in the patients with AML, we clearly determined its high expression in various AML cells. Studies have pointed to the involvement of BDH2 in the regulation of AML progression. High expression of BDH2 was related to shorter overall survival of AML patients, validating BDH2 as an independent prognostic marker for AML [16]. In this study, RAB27B could combine with BDH2. How RAB27B interact with BDH2 and how/whether this interaction affects the levels of RAB27B or BDH2 still require deeper exploration. Specifically, RAB27B silencing led to lower BDH2 expression, disclosing their positive-binding relationship. For further acquisition of the mechanism of action, BDH2 was overexpressed for the observation of changes in cell proliferation, cell cycle, and apoptosis of AML cells pretreated by si-RAB27B. As a consequence, si-RAB27B incurred decreased proliferation, enhanced cell cycle arrest and increased cell apoptosis, which was abrogated by BDH2 overexpression. Thus, it was the synergistic role of RAB27B and BDH2 that had influence on the cell behaviors of AML cells. Emerging evidences revealed that RAB27B is implicated in the migration of cancerous cells [29–31]. However, more research is needed to determine the role of RAB27B/BDH2 in migration of AML cells and confirm the mechanism of action of RAB27B/BDH2 in vivo.

## Conclusion

On the whole, we provide the first evidence that that RAB27B inhibits proliferation and promotes apoptosis of leukemic cells via interacting with BDH2, revealing that the RAB27B can functionally act as a prognostic marker of AML and targeting RAB27B may be conducive to the management of AML.

## Highlights

- Increased expression of RAB27B in acute myeloid leukemia is positively correlated with patients’ poor prognosis.
- RAB27B silencing suppressed AML cell proliferation and promoted G0/1 arrest and apoptosis.
- RAB27B interacts with BDH2 and regulates its expression.
- RAB27B is implicated in AML cell proliferation and apoptosis via BDH2.
